# Formation and Interfacial Behavior of Chitosan–Alginate Interpolyelectrolyte Complexes: From Bulk Dispersions to Layer-by-Layer Films

**DOI:** 10.3390/polym17223073

**Published:** 2025-11-20

**Authors:** Rafael Martín-López, Ana Puente-Santamaría, Ramón G. Rubio, Francisco Ortega, Eduardo Guzmán

**Affiliations:** 1 Departamento de Química Física, Facultad de Ciencias Químicas, Universidad Complutense de Madrid, Plaza de Ciencias s/n, Ciudad Universitaria, 28040 Madrid, Spain; rafmar05@ucm.es (R.M.-L.); anapuent@ucm.es (A.P.-S.); rgrubio@quim.ucm.es (R.G.R.); fortega@quim.ucm.es (F.O.); 2 Instituto Pluridisciplinar, Universidad Complutense de Madrid, Paseo Juan XXIII 1, 28040 Madrid, Spain

**Keywords:** alginate, chitosan, interfacial tension, interpolyelectrolyte complexes, layer-by-layer assembly, Quartz Crystal Microbalance, turbidity, zeta potential

## Abstract

This study investigates the formation, physicochemical properties, and interfacial behavior of interpolyelectrolyte complexes (IPECs) composed of chitosan (CS) and sodium alginate (ALG) in aqueous media at pH 4.5. Using a combination of turbidity, ζ-potential, conductivity, and interfacial tension measurements, we explore how mixing protocols and solution composition influence complex formation and stability. The results reveal that while ζ-potential remains largely unaffected by polymer concentration, turbidity and interfacial tension exhibit strong dependence, particularly near the stoichiometric charge equivalence point (*Z* ≈ 1). These findings suggest that neutral complexes formed at *Z* ≈ 1 display enhanced aggregation and surface activity, especially when ALG is in excess. Additionally, we extend the study to layer-by-layer (LbL) films assembled from CS and ALG, monitored via Quartz Crystal Microbalance with dissipation (QCM-D). The films exhibit quasi-linear growth and increasing elastic modulus with layer number, indicating uniform deposition and strong interlayer interactions. The viscoelastic properties of the multilayers further confirm the structural integrity and potential applicability of these systems in surface engineering and encapsulation technologies. Overall, this work provides a comprehensive understanding of CS–ALG complexation from bulk to interfacial assemblies.

## 1. Introduction

Interpolyelectrolyte complexes (IPECs), formed through the electrostatic-driven association of oppositely charged polymers constitute a versatile class of soft materials whose properties can be finely tuned by controlling pH, ionic strength, stoichiometry, and molecular architecture. These systems have received growing attention in recent years due to their inherent biocompatibility, environmental responsiveness, and ability to generate dispersed aggregates, gels, and multilayer films with diverse structural and functional characteristics. As a result, IPECs have found increasing relevance across biomedical, environmental, and materials science applications [1,2,3,4]. Among the many IPECs studied to date, complexes based on naturally occurring polysaccharides are particularly appealing. Their renewability, biodegradability, low toxicity, and structural diversity make them promising candidates for applications in drug delivery, regenerative medicine, food technology, and environmental remediation [5,6,7]. It is important to note that the formation of IPECs is influenced by several factors, including pH, ionic strength, mixing protocol, and polymer concentration [8,9,10]. These parameters affect not only the stability and morphology of the complexes but also their charge compensation and aggregation behavior, which are critical for applications requiring colloidal stability or interfacial activity.

Among polysaccharides, chitosan (CS) and sodium alginate (ALG) are considered two of the most widely studied. CS is CS, a cationic polysaccharide obtained by deacetylation of chitin, exhibits pH-dependent solubility and mucoadhesive behavior, while ALG, an anionic copolymer of mannuronic and guluronic acids, forms viscous solutions and gels through electrostatic and ion-mediated interactions. Their combination leads to CS–ALG complexes with tunable charge, morphology, and mechanical properties, which have been exploited in controlled drug release [11,12,13,14,15], wound healing and tissue engineering [14,15], food packaging [16], and the sequestration of contaminants in water treatment [17,18].

Recent studies have emphasized the importance of stoichiometric charge ratio (*Z*) in determining the physicochemical properties of IPECs [19]. For instance, Schoeller et al. [20] demonstrated that pH-responsive chitosan (CS)–sodium alginate (ALG) complexes deposited on electrospun PLGA nanofibers exhibit controlled drug release behavior, with surface charge and interfacial interactions modulated by *Z*. Similarly, Wasupalli and Verma [21,22] studied the role of IPECs formed by CS and polygalacturonic acid in antimicrobial and mechanical performance enhancement across various applications. These findings highlight the critical importance of precisely controlling complexation conditions to tailor the functional properties of IPECs.

Based on the previous discussion, the occurrence of non-equilibrium phenomena, particularly those driven by Marangoni stresses during the mixing process, should not be ignored during the formation of IPECs [23]. Such stresses can induce convective flows and compositional heterogeneities during the early stages of complex formation. Although these effects have been extensively studied in analogous systems, such as mixtures of oppositely charged polyelectrolytes and surfactants [24,25,26,27], they remain largely unexplored in the context of IPEC assembly. Non-equilibrium mixing conditions can lead to transient concentration gradients and localized charge imbalances, ultimately resulting in colloidal dispersions with physicochemical properties that deviate significantly from those expected under equilibrium complexation. These deviations may manifest in altered particle size distributions, aggregation behavior, and interfacial activity, all of which are critical parameters for applications involving drug delivery, encapsulation, or surface modification. Evidence supporting the influence of mixing order and non-equilibrium effects in IPEC formation has been reported by Saether et al. [28], who investigated the complexation of CS and ALG. Their study demonstrated that the sequence of component addition markedly affects both the aggregation state and the ζ-potential of the resulting complexes, suggesting that kinetic factors and initial concentration gradients play a decisive role in determining the final structure and charge distribution. These observations underscore the need for a deeper understanding of the dynamic processes governing IPEC formation, particularly when designing systems for reproducible and scalable applications. Another underexplored yet critical aspect of IPECs lies in their interfacial properties, which play a pivotal role in applications involving emulsions, foams, and surface coatings [29,30,31]. The ability of IPECs to adsorb at fluid interfaces and modulate interfacial tension directly influences their performance in stabilizing dispersed systems and forming functional films. Despite their relevance, systematic investigations into the surface activity of IPECs remain limited, particularly in comparison to well-characterized surfactant–polyelectrolyte mixtures [24,32,33,34]. Understanding how factors such as stoichiometric ratio, polymer concentration, and mixing protocol affect interfacial behavior is essential for optimizing IPEC-based formulations in colloidal and interfacial technologies. In this context, our work builds directly upon foundational studies [28] and presents a systematic investigation that connects bulk dispersion behavior, interfacial activity, and Layer-by-Layer (LbL) assembly. The objective is to bridge these domains and provide a unified understanding of the physicochemical mechanisms governing the formation of polysaccharide-based IPECs both in solution and at interfaces. Emphasis is placed on elucidating the influence of mixing protocol, stoichiometric ratio, and polymer concentration. By integrating bulk dispersion analysis with LbL film characterization, this study offers a comprehensive view of chitosan–alginate (CS–ALG) interactions across multiple scales, contributing to the rational design of functional polyelectrolyte systems. Moreover, a deeper understanding of the formation and interfacial behavior of CS–ALG complexes is crucial for optimizing bio-based technologies such as controlled drug delivery, encapsulation of active molecules, and wastewater remediation [20,35,36].

## 2. Materials and Methods

### 2.1. Chemicals

Chitosan (CS), a cationic polysaccharide with an average molecular weight ranging from 100 to 300 kDa and a degree of deacetylation of 90%, was obtained from Thermo Fisher Scientific (Waltham, MA, USA). Sodium alginate (ALG), an anionic polysaccharide with an average molecular weight between 12 and 40 kDa, was purchased from Aldrich (Darmstadt, Germany).

Glacial acetic acid (ReagentPlus, purity ≥ 99%) and sodium hydroxide (purity ≥ 99%) were used for pH adjustment and were supplied by Aldrich (Darmstadt, Germany). All solutions were prepared using ultrapure deionized water of Milli-Q grade (resistivity > 18 MΩ·cm; total organic carbon < 6 ppm), obtained from an AquaMAX™-Ultra Series 370 multicartridge purification system (Young Lin Instrument Co., Ltd., Anyang, Republic of Korea).

### 2.2. Preparation of Polysaccharide Solutions

The preparation of CS and ALG solutions at pH 4.5 was carried out following the protocol established in previous studies [37,38]. Appropriate amounts of CS or ALG were weighed to prepare stock solutions at a concentration of 50 mM and transferred into 250 mL flasks. Each flask was partially filled with deionized water, followed by the addition of 100 μL of glacial acetic acid to lower the pH. The mixtures were stirred continuously for 24 h to ensure complete dissolution. Subsequently, the pH of each solution was carefully adjusted to 4.5 by the dropwise addition of a 10^−2^ mM sodium hydroxide solution. The final volume was reached by adding an aqueous solution of glacial acetic acid pre-adjusted to pH 4.5.

### 2.3. Preparation of CS-ALG Mixtures

The interpolyelectrolyte complex (IPEC) dispersions were prepared using a mixing protocol adapted from the method developed by Ravera et al. [39], originally designed for particle–surfactant systems. In this approach, equal volumes of CS and ALG solutions (both adjusted to pH 4.5) were combined to form the IPEC dispersions. Specifically, one of the polysaccharide solutions, prepared at twice the target final concentration (2 mM or 0.2 mM), was added dropwise to the other polysaccharide solution, also at twice the desired final concentration but with variable composition. This stepwise addition was implemented to minimize concentration gradients during mixing and promote uniform complex formation [24,40,41].

The resulting dispersions were stirred at 1000 rpm for 30 min to ensure homogeneity, followed by overnight equilibration under static conditions. Since acetic acid does not act as a buffer, pH measurements were performed both after the aging period and immediately prior to sample use to confirm stable pH conditions around 4.5. This procedure was also applied to individual CS and ALG solutions.

### 2.4. Experimental Techniques

Turbidity (*τ*) of the CS–ALG mixtures was evaluated from absorbance measurements, according to the following expression,

(1) $$\tau =1-10^{-A},$$

where A denotes the measured absorbance of the sample. Spectrophotometric measurements were conducted at 450 nm using a Jasco FP-6500 spectrophotometer (Jasco Inc., Tokyo, Japan). This wavelength was chosen to minimize interference from the intrinsic absorption bands of the sample components.

The net charge of the formed complexes was evaluated by determining their electrophoretic mobility ($u_{e}$) via Laser Doppler Velocimetry, using a Zetasizer Nano ZS instrument (Malvern Instruments Ltd., Malvern, UK). The relationship between electrophoretic mobility and zeta potential (*ζ*) was established using Henry’s equation,

(2) $$u_{e}=\frac{2\varepsilon \zeta f(\kappa a)}{3\eta }.$$

In this equation, ε  and η represent the dielectric permittivity and viscosity of the medium, respectively, while f(κa) is the Henry function. For the present system, f(κa) was set to 1.5, consistent with the Smoluchowski approximation, as the particle size exceeds the thickness of the electrical double layer [42].

The ionic conductivity of the aqueous mixtures was determined using a Metrohm 856 Conductometer fitted with a platinum five-ring conductivity cell (Metrohm AG, Herisau, Switzerland).

Interfacial tension at the air/dispersion interface was measured using a K10T digital tensiometer (KRÜSS GmbH, Hamburg, Germany) equipped with a platinum Wilhelmy plate probe (contact area: 40.5 mm). Before each measurement, the probe was thoroughly cleaned with ethanol and Milli-Q water, followed by flame treatment with an ethanol torch to eliminate any residual organic contaminants. The dispersions were placed in glass cuvettes that were similarly cleaned with ethanol and Milli-Q water prior to use. All measurements were conducted at a controlled temperature of 25 °C, maintained using a thermostatic bath. Each reported value corresponds to the mean of at least three independent measurements. Measurements continued until a stable interfacial tension value was achieved.

A Quartz Crystal Microbalance with dissipation monitoring (QCM-D), model Explorer from QSense (Gothenburg, Sweden), was employed to investigate the adsorption behavior of polyelectrolyte–surfactant assemblies on negatively charged surfaces. Measurements were conducted using gold-coated AT-cut quartz crystals. Prior to use, the sensors were cleaned by immersion in a freshly prepared Piranha solution (70% H_2_SO_4_ and 30% H_2_O_2_) for 30 min, followed by extensive rinsing with Milli-Q water to remove residual contaminants. To impart a stable negative surface charge, the cleaned gold substrates were functionalized with a self-assembled monolayer of 3-mercapto-1-propane sulfonic acid (Aldrich, Darmstadt, Germany). QCM-D measurements were performed by recording the impedance spectrum of the quartz crystal at its fundamental resonance frequency (5 MHz) and at odd harmonics up to the 13th overtone (central frequency ~65 MHz). The resulting data were interpreted using a viscoelastic single-layer model, following the framework introduced by Voinova et al. [43]. This model relates shifts in frequency (Δ*f*) and energy dissipation (ΔD) across multiple overtones to key physical parameters of the adsorbed film, including thickness, viscosity, and elasticity.

## 3. Results and Discussion

### 3.1. Understanding the Formation of IPECs in Solution

**Figure 1:**
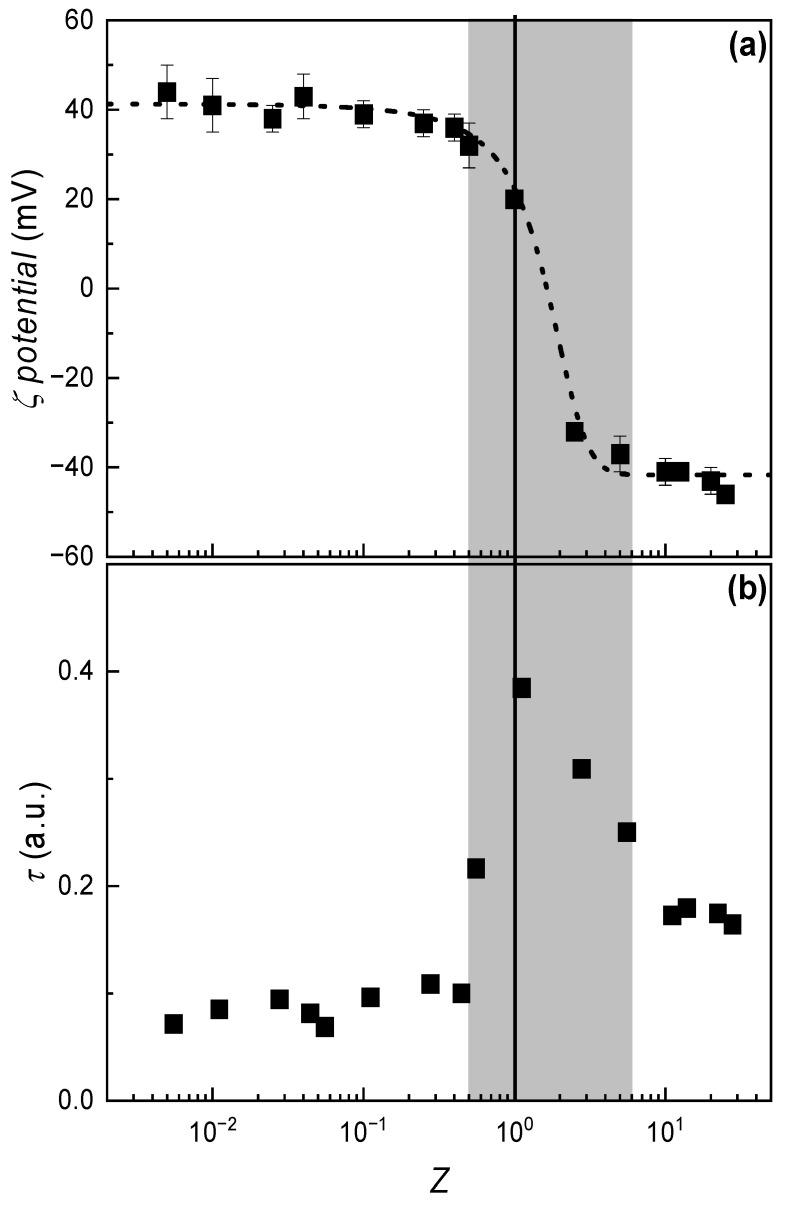
(**a**) Variation in ζ-potential as a function of the *Z* for IPEC dispersions formed by mixing equal volumes of a solution with a fixed concentration of CS with ALG solutions of varying concentrations. Symbols represent experimental data; the dashed line is included as a visual guide. The error bars represent the standard deviation of five independent measurements. (**b**) Turbidity variation as a function of *Z* for the same dispersions. Symbols represent experimental data, obtained as the average of five independent measurements with a standard deviation smaller than the size of the symbol. The vertical solid line corresponds to the composition *Z* = 1, while the shaded region indicates the biphasic zone.

The initial stage of this study focused on elucidating the formation mechanism of IPECs resulting from the interaction between CS and ALG at pH 4.5. In aqueous media, these oppositely charged polysaccharides exhibit a strong tendency to associate via electrostatic interactions, leading to the formation of stable complexes. To investigate this process, a fixed volume of CS solution at a constant concentration (2 mM, calculated based on the number of charge-bearing monomeric units) was added to ALG solutions of equal volume but varying concentrations. The evolution of complex formation was monitored by measuring the *ζ*-potential of the resulting dispersions as a function of the stoichiometric charge ratio (Z=nALGnCS, with $n_{ALG}$ and $n_{CS}$ being the mol number of ALG and CS in the mixture, respectively), providing insight into the electrostatic behavior and stability of the complexes (Figure 1a).

The *ζ*-potential data reveal a progressive shift in both magnitude and sign as *Z* increases. At *Z* < 1, the *ζ*-potential remains positive, indicating a predominance of positively charged chitosan monomers. As the concentration of ALG increases, a gradual decline in *ζ*-potential is observed. This behavior reflects the electrostatic association between CS and ALG monomers, resulting in a progressive neutralization of the system’s net charge. Charge inversion occurs when the number of negatively charged ALG monomers exceeds that of the positively charged CS monomers. While a strictly stoichiometric 1:1 association predicts inversion at *Z* = 1, the experimental data show that inversion occurs at a slightly higher *Z* value. This deviation is likely due to the nature of CS and ALG as weak polyelectrolytes, whose charge densities are pH-dependent. The pH was maintained at 4.5 to ensure optimal solubility and high charge density for CS. However, at this pH, partial protonation of ALG’s carboxylate groups may reduce its effective negative charge, thereby requiring a higher ALG concentration to achieve charge neutrality. Considering the pK_a_ values of ALG (≈3.4–3.7 depending on mannuronic acid/guluronic acid ratio [44]), approximately 10% of its carboxylate groups remain protonated at pH 4.5, which reduces the effective negative charge density and shifts the apparent charge inversion point to values of Z slightly above unity.

To further characterize the IPEC formation process, turbidity measurements were performed as a function of *Z* (Figure 1b). These results show a clear correlation with *ζ*-potential values and reveal three distinct behavioral regions. At both low and high Z values, turbidity remains low and relatively constant, corresponding to regions of constant *ζ*-potential. These zones are identified as monophasic (1*ϕ*), where the complexes are stabilized by an excess of either positive or negative charge. In contrast, near the point of charge inversion, a pronounced peak in turbidity is observed. This increase is attributed to the reduced net charge of the complexes, which promotes aggregation. As a result, solid particles form in the aqueous medium, giving rise to a biphasic system (2*ϕ*).

### 3.2. Influence of Mixing Procedure on the Formation of IPECs

**Figure 2:**
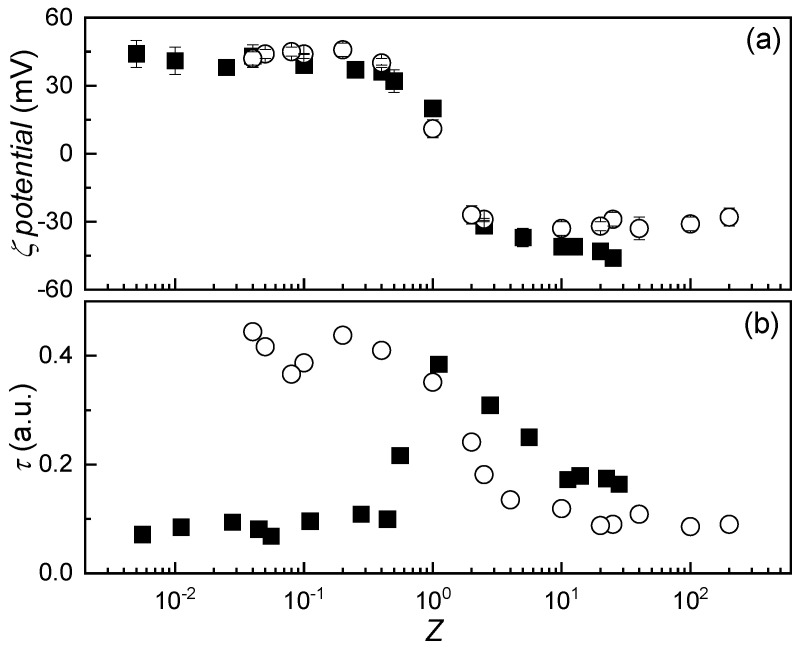
(**a**) Variation in ζ-potential as a function of *Z* for IPEC dispersions formed by adding a solution with a fixed concentration of CS (2 mM) to solutions with varying concentrations of ALG (■), or a solution with a fixed concentration of ALG (2 mM) to solutions with varying concentrations of CS (○). The error bars represent the standard deviation of five independent measurements. (**b**) Turbidity profiles of the same IPEC dispersions reported in panel (**a**) as a function of *Z*: fixed CS with variable ALG (■), and fixed ALG with variable CS (○). Symbols represent experimental data, obtained as the average of five independent measurements with a standard deviation smaller than the size of the symbol.

One of the longstanding challenges in the physicochemical study of systems formed by the association of charged species is the potential emergence of non-equilibrium phenomena during the mixing process. Such effects are often linked to Marangoni stresses, which arise from concentration heterogeneities of one or more components within the solution [24,25,26,27]. In the present study, the mixing protocol was specifically designed to minimize the influence of these non-equilibrium effects during the formation of IPECs. To assess whether these phenomena were effectively suppressed, experiments were conducted in which the order of component addition was systematically varied. This approach involved preparing mixtures with equivalent final compositions by either adding a fixed amount of chitosan to alginate solutions of varying concentrations or, conversely, adding a fixed amount of alginate to chitosan solutions with variable concentrations, both using equal volumes. Figure 2a presents the evolution of the *ζ*-potential of IPEC dispersions prepared under these different mixing sequences as a function of *Z*. The data show that the effective charge of the system remains largely unaffected by the order of addition, suggesting that the average charge distribution is preserved when the final composition is held constant. This outcome is consistent with the nature of *ζ*-potential measurements, which reflect the mean electrostatic properties of the dispersions. However, the absence of significant changes in *ζ*-potential alone does not conclusively rule out the presence of non-equilibrium effects during complex formation. To further investigate this possibility, turbidity measurements were performed on mixtures prepared with reversed addition sequences (see Figure 2b), offering complementary insight into potential structural or aggregation differences induced by mixing dynamics.

A comparative analysis of turbidity data reveals that the behavior of the system is notably influenced by the mixing protocol, particularly the order in which the components are combined. As discussed in Section 3.1, when chitosan CS is added to ALG solutions of varying concentration, the resulting turbidity profiles exhibit three distinct compositional regions, consistent with the expected charge distribution of the system. However, reversing the order of addition, i.e., introducing ALG into CS solutions, leads to a markedly different outcome. At high *Z* values (low CS content), the system displays low turbidity, indicative of a monophasic region (1*ϕ*), which aligns with the effective charge of the complexes. This behavior is consistent with previous observations for mixtures of equivalent composition but obtained using the inverse mixing protocol.

As the CS concentration increases (corresponding to decreasing *Z*), turbidity rises, reaching a maximum near *Z* = 1. This peak is consistent with phase separation occurring near the system’s isoelectric point. Interestingly, further addition of CS does not result in a return to low turbidity or the re-establishment of a monophasic region. Instead, the system maintains elevated turbidity levels, despite the composition suggesting a return to a 1*ϕ* regime. These findings indicate that the mixing order significantly impacts the structural properties of the complexes, in agreement with previous reports by Saether et al. [28] for IPECs and by Mészáros et al. [26] in mixtures of polyelectrolytes and surfactants bearing opposite charges. This behavior suggests the presence of non-equilibrium phenomena during complex formation. To understand the origin of these effects, several factors must be considered: (i) the mixing process, (ii) the sequence of component addition, and (iii) the molecular structure of CS and ALG. While the dropwise addition method is designed to minimize Marangoni gradients and promote uniform mixing, the interplay between mixing order and polymer structure appears to play a critical role. Both CS and ALG are polysaccharides, but they differ significantly in molecular weight. CS chains exhibit an average molecular weight approximately one order of magnitude higher than that of ALG. Consequently, at equal concentrations, CS solutions are more viscous than ALG solutions. This difference may affect the distribution of the added polymer during mixing. When ALG is introduced into a viscous CS solution, particularly at high CS concentrations, the elevated viscosity hinders uniform dispersion, leading to local compositional heterogeneities and promoting non-equilibrium association. In contrast, when CS is added to ALG, the lower viscosity of the receiving solution facilitates more homogeneous mixing, thereby minimizing non-equilibrium effects.

**Figure 3:**
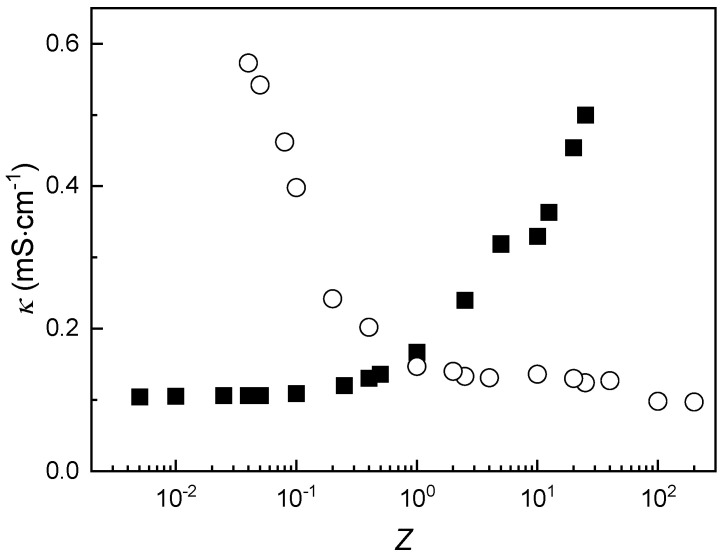
Variation in κ as a function of *Z* for IPEC dispersions formed by adding a solution with a fixed concentration of CS (2 mM) to solutions with varying concentrations of ALG (■), or a solution with a fixed concentration of ALG (2 mM) to solutions with varying concentrations of CS (○). Symbols represent the average of ten independent measurements with a standard deviation smaller than the size of the symbol.

It is worth noting that although the addition of CS to ALG appears to reduce non-equilibrium behavior, a detailed evaluation of this aspect requires monitoring the evolution of the samples over time [24]. Further insight into the association process can be gained by examining the specific conductivity (*κ*) of the IPEC dispersions as a function of composition. Figure 3 illustrates the variation in conductivity with *Z* for dispersions prepared by adding a fixed amount of CS (or ALG) to solutions of variable ALG (or CS) concentration.

The first aspect to consider from the specific conductivity data is the presence of two distinct compositional dependencies, regardless of the mixing protocol employed. When *Z* is low in mixtures prepared by adding CS to alginate ALG, or when *Z* is high in mixtures formed by adding ALG to CS, conductivity increases only slightly with changes in *Z* until approaching Z ≈ 1. At this point, a marked change in slope is observed in the conductivity profiles. This inflection is attributed to the complete neutralization of one polyelectrolyte’s charge through complexation with the other, followed by the emergence of excess charged monomers from the dominant component. These free ions contribute to the observed increase in conductivity. Notably, at the extremes of *Z*→0 and *Z*→∞, the conductivity values converge to those of the individual 1 mM CS and ALG solutions, measured at 0.104 mS·cm^−1^ and 0.090 mS·cm^−1^, respectively. These results confirm that, independent of the mixing sequence, the equivalence point for complex formation, where electrically neutral complexes are formed, occurs near *Z* = 1 under the experimental conditions. This finding aligns with previous observations reported by Argüelles-Monal et al. [45] for IPECs formed from chitosan hydrochloride and sodium polygalacturonate.

It is worth noting that although our measurements are steady-state, the observed asymmetry in turbidity and conductivity with mixing order represents indirect evidence of kinetic trapping and transient gradients during early-stage complexation, consistent with non-equilibrium behaviors reported in analogous oppositely charged systems [24,25,26,40]. It is clear that the use of time-resolved measurements may extend our understanding related to the emergence of non-equilibrium during the association, as was demonstrated for mixtures of oppositely charged polyelectrolytes and surfactants in previous studies [24,46]. However, considering the simultaneous binding for multiple sites during the formation of IPECs, the timescales involved in the equilibration may be relatively difficult to obtain feasible information on the equilibration dynamics of polyelectrolyte-surfactant mixtures.

### 3.3. Influence of Solution Composition on the Formation of IPECs

**Figure 4:**
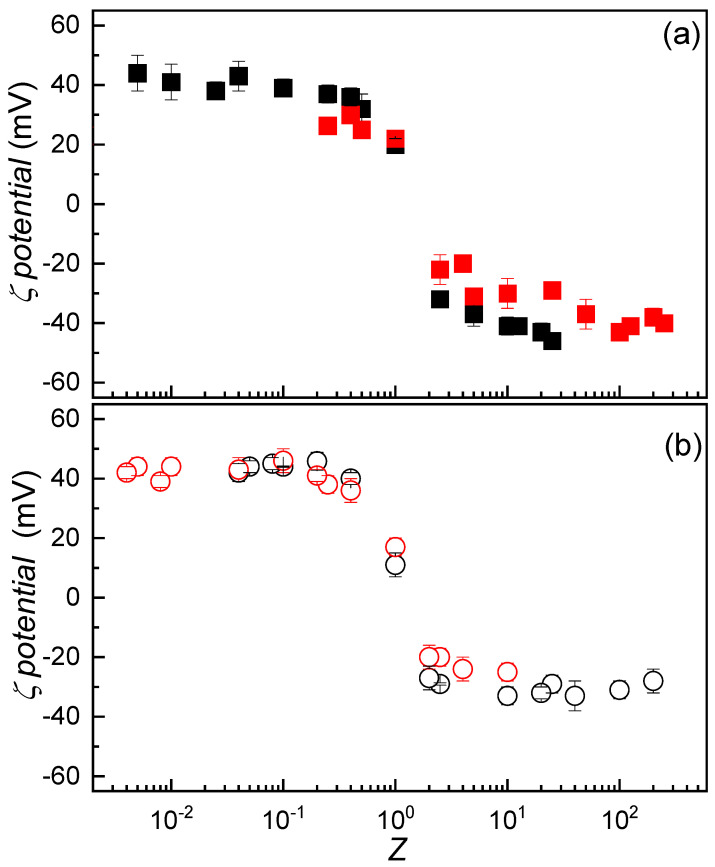
(**a**) *ζ*-potential as a function of the stoichiometric *Z* for IPEC dispersions formed by adding a solution of fixed CS concentration (■ 2 mM and ■ 0.2 mM) to ALG solutions of varying concentration. (**b**) *ζ*-potential as a function of *Z* for IPEC dispersions formed by adding a solution of fixed ALG concentration (○ 2 mM and ○ 0.2 mM) to CS solutions of varying concentration. In both panels, the error bars represent the standard deviation of five independent measurements.

The discussion presented in the preceding sections has clarified several key aspects of the systems under investigation, including the mechanism of IPEC formation and the potential role of non-equilibrium phenomena in this process. In this section, we examine how the composition of the initial solutions influences the formation of IPECs. To explore this effect, an approach analogous to that described in Section 3.3 was employed. Figure 4a illustrates the variation in *ζ*-potential as a function of *Z* for dispersions prepared by adding fixed amounts of CS to ALG solutions of constant volume but varying concentration.

**Figure 5:**
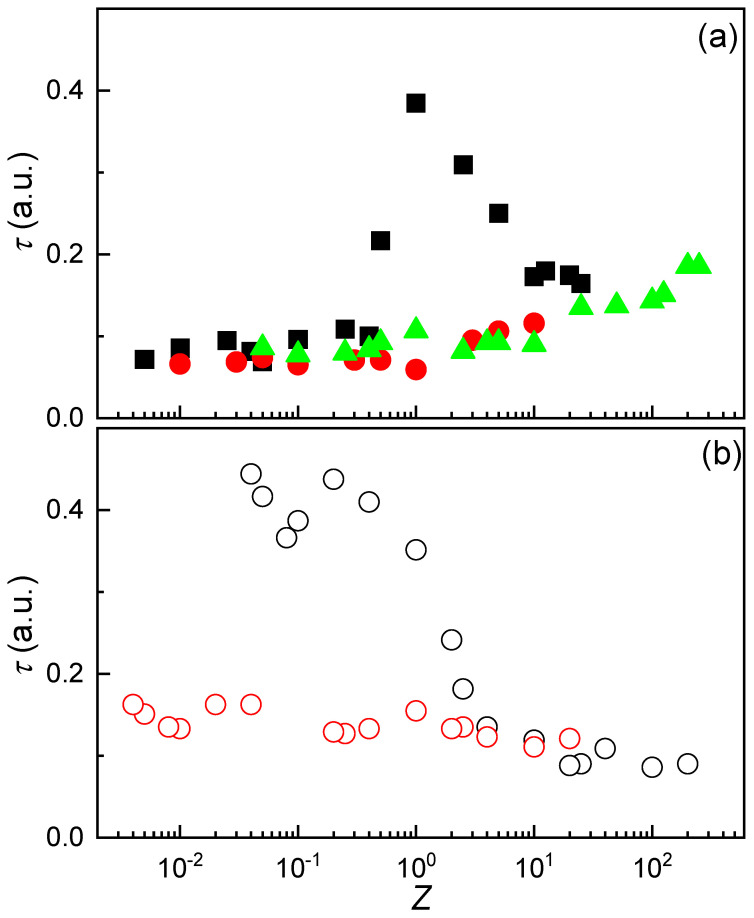
(**a**) Turbidity as a function of *Z* for IPEC dispersions formed by adding a solution of fixed CS concentration (■ 2 mM, ▲ 0.5 mM and ● 0.2 mM) to ALG solutions of varying concentration. (**b**) Turbidity as a function of *Z* for IPEC dispersions formed by adding a solution of fixed ALG concentration (○ 2 mM and ○ 0.2 mM) to CS solutions of varying concentration. In both panels, symbols represent the average of five independent measurements with a standard deviation smaller than the size of the symbol.

The variation in *ζ*-potential with dispersion composition was found to be consistent regardless of the initial concentration of the CS solution used. A similar trend was observed when dispersions were prepared by adding a fixed concentration of ALG to CS solutions of varying concentration (see Figure 4b). Given that the initial concentration of the solutions used for IPEC preparation does not significantly influence the effective charge of the system, it becomes relevant to explore how turbidity responds to changes in the concentration of the starting solutions. Figure 5a presents the turbidity profiles as a function of the stoichiometric charge ratio (Z) for mixtures prepared by adding CS solutions at three different concentrations (0.2 mM, 0.5 mM, and 2 mM) to fixed volumes of ALG solutions with variable concentration.

Unlike the *ζ*-potential, which remained largely unaffected by the concentration of the added polymer solution, turbidity exhibited a pronounced dependence on this parameter. When CS or ALG solutions at 2 mM concentration were used, a distinct increase in turbidity was observed near the stoichiometric charge equivalence point (*Z* ≈ 1). This behavior is attributed to the formation of larger neutral aggregates resulting from the association of oppositely charged species. Despite their electrical neutrality, these aggregates remained dispersed due to their high hydrophilicity, which is likely driven by the abundance of hydroxyl groups on their surfaces. In contrast, when the concentration of the added polymer solution was reduced, the turbidity profile changed significantly. Instead of a sharp transition near *Z* ≈ 1, turbidity increased gradually and monotonically, either with increasing *Z* when CS was added (Figure 5a), or with decreasing *Z* when ALG was added (Figure 5b). This absence of a distinct turbidity peak near charge equivalence may be explained by a reduced number of IPECs formed under lower polymer concentration conditions. Although the resulting complexes are electrically neutral, their lower abundance in the dispersion limits coalescence, thereby preventing a sharp increase in turbidity.

**Figure 6:**
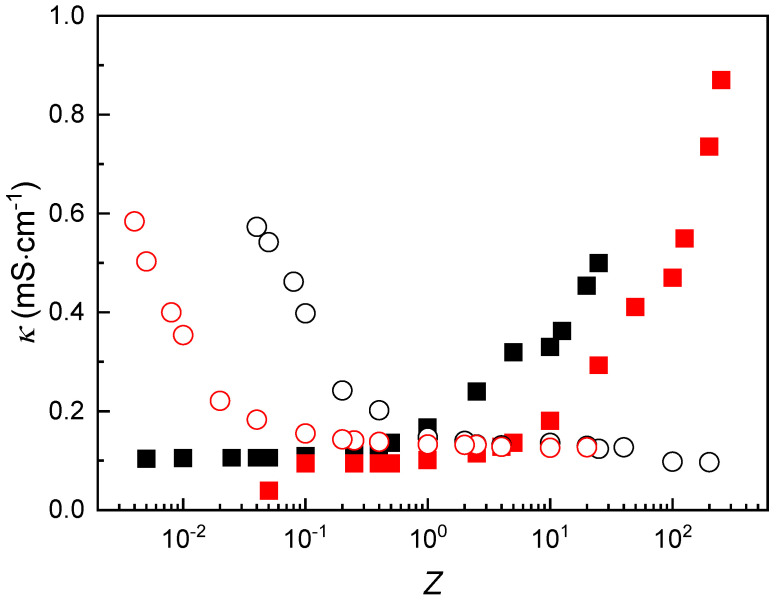
*κ* as a function of *Z* for IPEC dispersions formed by mixing fixed concentrations of CS (■ 2 mM and ■ 0.2 mM) with ALG solutions of varying concentration, or fixed concentrations of ALG (○ 2 mM and ○ 0.2 mM) with CS solutions of varying concentration. Symbols represent the average of ten independent measurements with a standard deviation smaller than the size of the symbol.

The continuous rise in turbidity with varying *Z* can be interpreted as a consequence of the presence of uncomplexed polymer segments within the IPECs for *Z* ≠ 1. These segments contribute to the overall optical density of dispersion, increasing the turbidity as *Z* assumes values far from unity. To further investigate the impact of polymer concentration on complex formation, the specific conductivity of the dispersions was analyzed as a function of composition and mixing protocol. The results are presented in Figure 6.

The specific conductivity profiles of the IPEC dispersions as a function of *Z* exhibit qualitatively similar trends regardless of the initial concentrations of the polymer solutions used. This consistent behavior reinforces the scenario previously discussed in Section 3.2 (see Figure 3 and its corresponding discussion). However, the absolute conductivity values are clearly influenced by the concentration of the added polymer. Specifically, reducing the concentration of the added CS or ALG solution causes a shift in the conductivity curve: toward lower *Z* values when ALG is added, and toward higher *Z* values when CS is added. This shift can be rationalized by considering the concentration-dependent formation of complexes in the aqueous medium. A lower concentration of the added polymer results in fewer IPECs being formed, which in turn reduces the number of charge carriers in the system. Consequently, the overall specific conductivity of the dispersion decreases.

### 3.4. Adsorption of IPECs to the Air/Dispersion Interface

**Figure 7:**
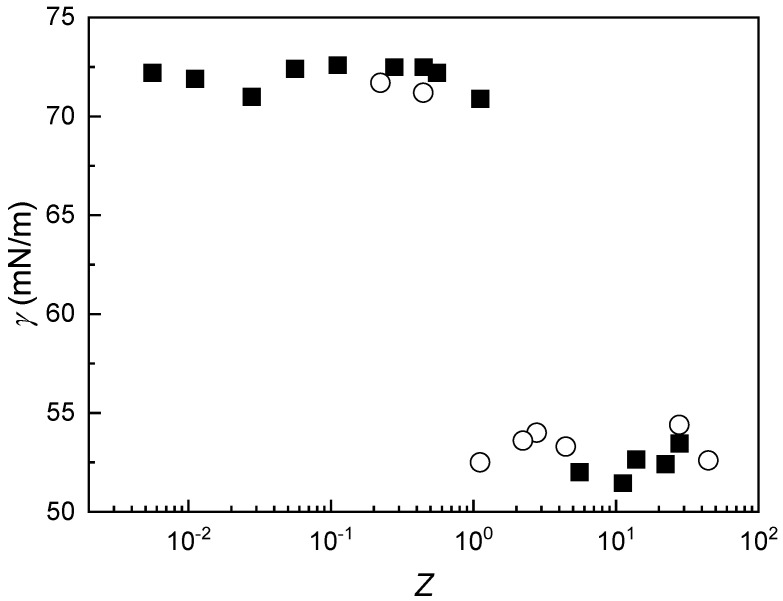
Variation in γ as a function of *Z* for IPEC dispersions formed by adding a solution with a fixed concentration of CS (2 mM) to solutions with varying concentrations of ALG (■), or a solution with a fixed concentration of ALG (2 mM) to solutions with varying concentrations of CS (○). Symbols represent the average of three independent measurements with a standard deviation smaller than the size of the symbol.

Given the similarities between IPECs and polyelectrolyte–surfactant complexes, and considering the surface-active nature of the latter [47], the ability of IPECs to adsorb at the air–water interface was evaluated through interfacial tension (*γ*) measurements. Figure 7 presents the interfacial tension profiles as a function of Z for complexes prepared using both mixing methodologies discussed earlier.

In contrast to surfactant solutions or polyelectrolyte–surfactant mixtures [24,47,48], which typically exhibit a monotonic variation in surface tension with composition, the behavior observed in IPEC dispersions is markedly different. For these systems, the stoichiometric charge equivalence point (*Z* ≈ 1) corresponds to a composition where the interfacial tension undergoes a sharp transition. When *Z* < 1, the interfacial tension of the IPEC dispersions remains close to that of pure water at 25 °C (~72 mN·m^−1^), indicating negligible surface activity for complexes with excess chitosan. In contrast, when *Z* > 1, the interfacial tension drops significantly to approximately 50 mN·m^−1^, suggesting that complexes with excess alginate exhibit pronounced surface activity.

**Figure 8:**
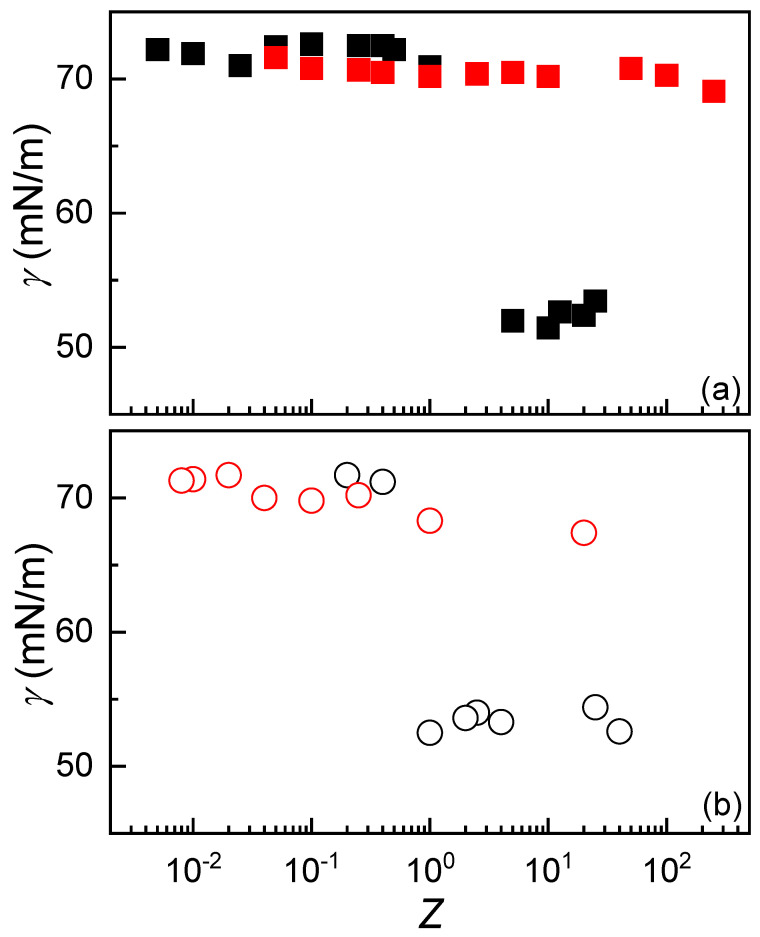
(**a**) Interfacial tension as a function of *Z* for IPEC dispersions formed by adding fixed concentrations of CS (■ 2 mM and ■ 0.2 mM) to ALG solutions of varying concentration. (**b**) Interfacial tension as a function of *Z* for IPEC dispersions prepared by adding fixed concentrations of ALG (○ 2 mM and ○ 0.2 mM) to CS solutions of varying concentration. In both panels, symbols represent the average of three independent measurements with a standard deviation smaller than the size of the symbol.

Following the same concept used in Section 3.3, the effect of the concentration of the added polymer solutions on the interfacial tension of IPEC dispersions was also investigated. Figure 8 illustrates the dependence of interfacial tension on the composition of the dispersions, expressed in terms of the stoichiometric charge ratio *Z* for dispersions obtained under different mixing conditions.

The interfacial tension results reveal significant differences depending on the concentration of the added polymer solution, particularly in relation to the interfacial activity of the resulting IPECs. When CS or ALG solutions at 2 mM concentration were used during complex formation, the charge equivalence region (*Z* ≈ 1) marked a clear transition between IPECs with negligible surface activity (*Z* < 1) and those exhibiting relatively high interfacial activity (*Z* > 1). However, upon reducing the concentration of the added CS or ALG solutions, the surface activity largely disappears. Only at high *Z* values is a slight decrease in interfacial tension observed, likely due to limited adsorption of IPECs at the air–water interface. This concentration-dependent behavior can be explained by considering that interfacial tension reduction generally arises from the packing of surface-active species, here, the IPECs, at the interface, along with lateral interactions between adsorbed particles. Therefore, lowering the polymer concentration in the dispersion reduces the number of IPECs available for adsorption, resulting in a lower interfacial particle density and consequently higher interfacial tension.

### 3.5. Assembly of LbL Films by Alternate Deposition of CS and ALG on Flat Surface

**Figure 9:**
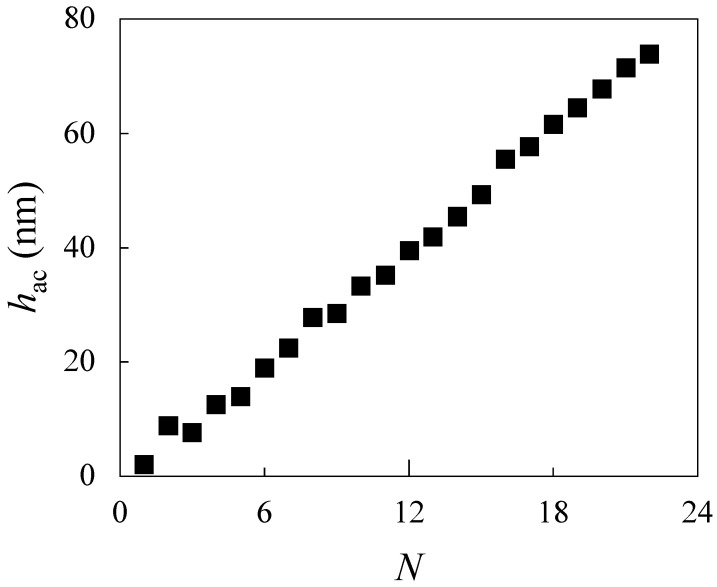
$h_{\mathrm{ac}}$ as a function of *N* for multilayer films formed via LbL assembly of CS and ALG, measured by QCM-D. Symbols represent the average of three independent measurements with a standard deviation smaller than the size of the symbol.

To complement our investigation of IPECs formed in bulk solution, we extended the study to examine the same polyelectrolyte pair, CS and ALG, within a layer-by-layer LbL assembly framework. While bulk complexation offers valuable insight into the intrinsic interactions and phase behavior of the system, the LbL technique provides a more controlled and spatially resolved approach to probing these interactions at interfaces [15,16]. Figure 9 presents the evolution of acoustic thickness ($h_{\mathrm{ac}}$) as a function of the number of adsorbed layers (*N*), as determined by QCM-D.

The data reveal a quasi-linear growth profile, indicating that the multilayer assembly proceeds in a uniform and reproducible manner. This behavior suggests that the polysaccharides are deposited homogeneously throughout the film. Such linearity in growth may be attributed to the relatively rigid nature of the polyelectrolyte chains, which likely adsorb onto the substrate in an extended, rod-like conformation. This minimizes surface roughness and promotes consistent layer formation. Additionally, the observed growth regime is consistent with a diffusion-limited adsorption mechanism and homogeneous charge compensation across the multilayer structure [49]. Moreover, the quasi-linear multilayer growth observed by QCM-D likely reflects the near-stoichiometric association and the strong interactions between the oppositely charged polysaccharides observed in bulk IPECs, indicating that similar electrostatic compensation governs both dispersed and adsorbed structures. This observation is consistent with the recent theoretical work of Subbotin and Semenov [50], who proposed that the most compact and stable multilayers are formed near stoichiometric complexation, as the same balance between electrostatic and short-range interactions controls both bulk IPEC organization and interfacial film buildup.

**Figure 10:**
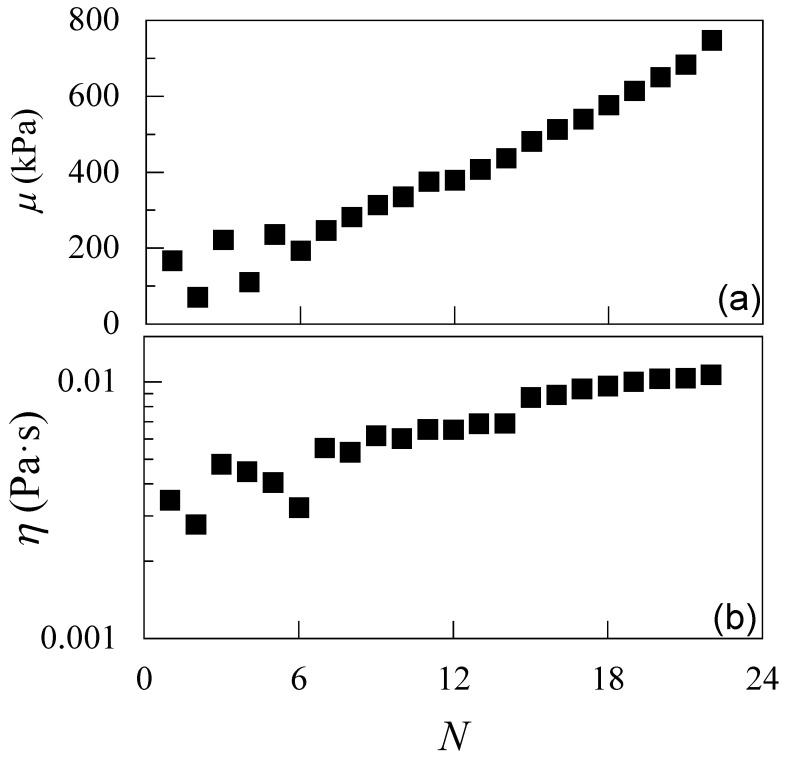
(**a**) Evolution of the average elastic shear modulus as a function of the number of deposited layers in LbL films composed of CS and ALG. The curve exhibits a peak near *N* ≈ 12, indicating a transition in the mechanical rigidity of the multilayer structure. (**b**) Average shear viscosity versus *N*. In both panels, symbols represent the average of three independent measurements with a standard deviation smaller than the size of the symbol.

The results obtained for the growth are compatible with the mechanical properties also obtained from the analysis of the QCM-D data (see Figure 10). In fact, the multilayer presents a relatively rigid structure, with the elastic shear modulus (*μ*) increasing with the number of deposited layers, whereas the viscosity (*η*) remains relatively low. This confirms the strong interaction between the adsorbed layers.

## 4. Conclusions

This work has been focused on the physicochemical characterization of interpolyelectrolyte complexes (IPECs) formed by chitosan (CS) and sodium alginate (ALG) in aqueous media at pH 4.5, with a particular emphasis on their bulk behavior, interfacial properties, and layer-by-layer (LbL) assembly. Through systematic variation in mixing protocols and solution compositions, we have elucidated key aspects of complex formation, offering insights relevant to both fundamental understanding and practical applications.

The formation of IPECs was shown to be strongly influenced by the stoichiometric charge ratio (*Z*), with *ζ*-potential and turbidity measurements revealing distinct monophasic and biphasic regimes. Near *Z* ≈ 1, the system approaches charge neutrality, leading to aggregation and phase separation, as evidenced by a pronounced turbidity peak. The *ζ*-potential data confirmed the occurrence of charge inversion, although the exact equivalence point deviated slightly from *Z* = 1 due to the pH-dependent ionization of the weak polyelectrolytes employed in the assembly of the IPEC. These findings underscore the importance of controlling solution pH and composition to modulate complex stability and aggregation behavior. On the other hand, the mixing protocol, specifically, the order of component addition, was found to significantly affect the structural properties of the resulting complexes. While *ζ*-potential remained largely unaffected, turbidity and conductivity profiles revealed notable differences depending on whether CS or ALG was added first. These effects were attributed to differences in solution viscosity and molecular weight, which influence the homogeneity of mixing and the likelihood of non-equilibrium association. The results highlight the need for careful control of mixing dynamics in the preparation of IPECs, particularly when targeting reproducible and scalable formulations.

Interfacial tension measurements demonstrated that IPECs exhibit surface activity only under specific compositional conditions. Complexes formed with excess ALG (*Z* > 1) showed a marked reduction in interfacial tension, suggesting their potential utility in stabilizing foams and emulsions. In contrast, complexes with excess CS (*Z* < 1) displayed negligible surface activity, with interfacial tension values close to that of pure water. The concentration of the added polymer solutions also played a critical role: higher concentrations promoted surface activity near *Z* ≈ 1, while lower concentrations suppressed it, likely due to reduced particle density at the interface. These observations provide valuable guidance for designing IPEC-based systems with tailored interfacial properties.

The study was further extended to the assembly of multilayer films via LbL deposition of CS and ALG on solid substrates. QCM-D measurements revealed a quasi-linear growth of acoustic thickness with the number of layers, indicating uniform and reproducible adsorption. Viscoelastic modeling confirmed that the multilayers possess increasing rigidity with layer number, consistent with the formation of compact and stable films. These results reinforce the theoretical view that multilayer films can be regarded as spatially constrained coacervate structures, where bulk charge compensation and interfacial activity directly dictate film architecture. In addition, our work demonstrates that the same polyelectrolyte pair can be effectively used to construct well-defined interfacial architectures, complementing their behavior in bulk dispersions.

Although the present study focuses on the chitosan–alginate system, the trends identified, such as the dependence of aggregation and interfacial activity on stoichiometric ratio and mixing order, are expected to be broadly applicable to mixtures including other oppositely charged polyelectrolytes as occurs in mixtures of oppositely charged polyelectrolytes and surfactants. Quantitative differences may arise due to variations in molecular weight, charge density, and ionization behavior.

Overall, this work advances our understanding of CS–ALG complexation across multiple scales, from dispersed particles to structured films, and highlights the interplay between composition, mixing protocol, and interfacial dynamics. The insights gained here lay the groundwork for future applications of IPECs in areas such as encapsulation, drug delivery, and surface functionalization, where control over charge, stability, and interfacial behavior is essential. These findings not only advance the fundamental understanding of CS–ALG complexation but also provide design principles for the development of biocompatible delivery, coating, and environmental remediation systems.

## Data Availability

The raw data supporting the conclusions of this article will be made available by the authors on request.
